# Synergic Improvement of Elastocaloric Effect in Polycrystalline NiMnGaCo by Magneto-Mechanical Coupling

**DOI:** 10.3390/ma19183842

**Published:** 2026-09-09

**Authors:** Francesca Villa, Corrado Tomasi, Francesca Passaretti, Nicola Bennato, Enrico Bassani, Emanuele Bestetti, Elena Villa

**Affiliations:** 1 CNR ICMATE, Lecco Unit, 23900 Lecco, Italy; francesca.villa@cnr.it (F.V.); francesca.passaretti@cnr.it (F.P.); nicola.bennato@cnr.it (N.B.); enrico.bassani@cnr.it (E.B.); emanuelebestetti@cnr.it (E.B.); 2 CNR ICMATE, Genova Unit, 16149 Genova, Italy; corrado.tomasi@cnr.it

**Keywords:** FeSMA, multicaloric effect, NiMnGaCo, multi-field coupling, magneto-mechanical coupling test

## Abstract

**Highlights:**

- Experimental investigation of magneto-mechanical coupling at different temperatures.
- Investigation of caloric effect from different points of view: ΔS and ΔT_ad_ vs. magnetic field.
- Magnetic field effect characterized by a magnetostrictive approach.
- Improvement of the efficiency of the elastocaloric effect with an increase in ΔT_ad_ of about 25%.

**Abstract:**

Among the ferromagnetic shape memory alloys (SMAs), Ni43Mn31Ga19Co7 (at%) alloys are well known as a promising ferromagnetic SMA (FeSMA) for magnetocaloric effect, and scientific interest has increased due to the possible exploitation of multicaloric function. This first example of multi-effect by coupling elastocaloric and magnetocaloric effect is still far from complete experimental validation, mainly due to the difficulty of testing conditions that require the simultaneous application of both fields. In this study, a low magnetic field (μ_0_H = 0.26 T and 0.56 T) is applied during elastocaloric deformation at different temperatures. Although the magnetic field values applied in this work are much lower than those usually considered in the magnetocaloric effect, the magnetic field improves the elastocaloric effect, increasing the total strain and reducing the critical stress and mechanical hysteresis. This synergic action allows us to obtain an increase in the ΔT_ad_ of about 13% and 25% upon loading and unloading, respectively.

## 1. Introduction

During the last few decades, Ni-Mn-Ga-based Heusler shape memory alloys (SMAs) have been widely investigated because of their martensite phase properties and, chiefly, for the strong coupling between magnetic and elastic degrees of freedom, as well as their very low twinning stress that allows the magnetic shape memory (MSM) effect or magnetic-field-induced strains (MFISs) [1,2,3,4,5,6,7,8,9,10,11]. Owing to these properties, they are potentially applicable in the fields of energy harvesting, refrigeration, actuators and sensors [12,13]. In particular, with regard to refrigeration, solid-state cooling technology driven by magnetic fields and mechanical stresses would allow the manufacture of eco-friendly devices and reduce environmental issues related to the current technology based on the employment of greenhouse and harmful gases. In this context, reducing emissions and developing environmentally sustainable technologies represent key factors for the transition towards a more sustainable and energy-efficient society.

The addition of Co to the Ni-Mn-Ga crystal structure leads to high temperature and less stable ferromagnetic shape memory alloys, particularly with low doping levels [14]. It is widely demonstrated that its presence increases the Curie temperature (TC), whereas the thermoelastic martensite transformation (TMT) temperature could rise or decrease with Co doping depending on the involved sublattice. It has been reported by some authors that replacement of Ni with Co produces a lowering in martensitic transition temperature, while Mn or Ga substitutions yield to its increase [15,16,17,18,19]. These possibilities of tuning the TMT temperature by replacement with Co add interest to using NiMnGaCo alloys for caloric purposes. In fact, Co alloying in Mn-rich Ni-Mn-Ga Heusler alloys deeply affects the exchange interactions of both austenite and martensite; hence, by adjusting the cobalt content, it is possible to switch from direct to inverse magnetocaloric effect [20,21]. The inverse magnetocaloric effect was also described by Gomes et al. [22] for Ni_2_Mn_1−x_Co_x_Ga (x = 0.10, 0.20, 0.30), while a direct effect was shown by Kaštil et al. in Er-conditioned Ni_42.9_Co_7.5_Mn_31.4_Ga_18.2_ alloy, where the presence of erbium plays a key role on magnetic properties of this alloy [23]. Moreover, as reported in the literature, different attempts were carried out on substitution of Co combined to Fe, Cu or In [24,25,26,27,28,29,30,31,32,33,34,35,36,37]. In these studies, the attention was mainly devolved to structural and magnetic–physical–chemical features, depending principally on the electronic distribution. Considering only Co addition, it is well established that Co alloying improves the mechanical properties of Ni-Mn-Ga SMAs. In fact, not only does it stabilize the thermoelastic transformation, but it also enables the brittleness of the materials to be reduced, which is one of the main limitations of the practical application of these kinds of alloys. In this frame, various investigations have focused their attention on the mechanical properties of Co-doped Ni-Mn-Ga alloys such as stress vs. strain response [13,17,38], cavitation resistance in composites, also in the high entropy field [39,40], as well as magnetostriction measurements [41,42], but, to our knowledge, little attention has been focused on elastocaloric assessments. Based on the above-mentioned features, the Co-doped Ni-Mn-Ga Heusler alloys attracted our interest as a multicaloric system because of the coupling between the magnetocaloric and the elastocaloric effects. This coupling produces appreciable effects even at low magnetic fields, whereas a significant magnetocaloric response could be reached only for higher field values (>5 T). In fact, for this system, we can consider the magnetocaloric effect as slightly different from the usual magnetocaloric effect in SMAs, where the entropy change related to the introduction of magnetic order is overlapped with the entropy change due to the phase transition between paramagnetic austenite and ferromagnetic martensite. In this case, the two phases are both ferromagnetic; therefore, the magnetocaloric effect is reduced to the change in magnetic order of the two phases. In this work, we propose a new experimental approach by presenting a mechanical study under a magnetic field. Such an approach enables us to evaluate this weak magnetocaloric effect coupled with the elastocaloric effect in Co-doped Ni-Mn-Ga alloys, which can be seen principally as a magnetic enhancement of the elastocaloric effect or improvement due to the modification of the mechanical path thanks to the magnetic effect. Therefore, we describe the action of the magnetic field in terms of magnetostrictive properties as well as a direct estimation of caloric efficiency expressed by ΔT_ad_.

## 2. Materials and Methods

The samples investigated are polycrystalline ferromagnetic Ni_43_Mn_31_Ga_19_Co_7_ (at%). Stoichiometric amounts of pure metal components (Ni 99.95%, Mn 99.7%, Ga 99.99% and Co 99.95%) were melted in an arc furnace (Leybold LK6/45, Cologne, Germany) in a slightly overpressured Ar static atmosphere, and the buttons were remelted to achieve homogenization of the material. Then the samples were heat-treated at 1173 K for 5 h in inert Ar static atmosphere: the samples were inserted into the furnace at 1173 K and cooled after 5 h by water quenching. Scanning electron microscopy (SEM) LEO 1430 (Zeiss, Oberkochen, Germany) analysis by means of energy-dispersive X-ray (EDX) spectroscopy confirmed the homogeneity of the alloy, the correspondence with nominal composition and the absence of secondary phases as well as the XRD preliminary control. For the purposes of this work, we consider the optical microscopic observation interesting, as it gives us a first overview of the grain structure. All the pictures were registered after chemically etching with Marble’s reagent for 30 s. Differential scanning calorimetric (DSC) analysis and thermo-magnetic analysis were performed for the assessment of thermoelastic martensitic (TMT) and magnetic transformations (MT). Calorimetric analysis was carried out by DSC Q100 (TA Instruments, New Castle, DE, USA) equipped with a liquid nitrogen cooling system at a rate of 10 K/min, Thermomagnetic analysis was carried out by the measurement of the a.c. (f = 500 Hz) magnetic susceptibility as a function of temperature in an applied field of 5 mT and 1.5 T. The set-up for magneto-mechanical coupling has already been described in detail [43], where magnetic fields of 0.26 T and 0.56 T can be applied by a permanent magnet in the longitudinal direction. The permanent magnets are stable until 250 °C. The magnetic field intensity produced was measured by Hall probe, and the values indicated are registered data along a length of about 10 mm. The measurements were performed in an E3000 Instron mechanical test instrument (Instron, Norwood, MA, USA) equipped with a load cell of 3 kN and a thermal chamber for precise control of the isothermal conditions. Further K-thermocouples were used to verify the programmed temperature had been reached and the stability of the isothermal conditions. Once it had been checked that the test temperature had been achieved, the mechanical measurements were conducted in compression configuration, in stress control, with a rate of 20 MPa/min, in isothermal conditions in the temperature range [418 K–448 K]. The adiabatic temperature change ΔT_ad_ measurements were carried out at 418 K for 10 cycles with a strain rate of 600%/min in 0 T and 0.56 T applied magnetic fields. The strain rate to reach the quasi-adiabatic condition was chosen with reference to our previous experimental investigation on an FeSMA specimen of similar size. We inserted a holding time of 60 s between one deformation cycle and the next to allow the sample to exchange heat with the surroundings until thermal equilibrium was reached. The control of the chamber temperature was carried out by a K-type thermocouple. The direct measurement of ΔT_ad_ was carried out on samples of 3 × 3 × 8 mm^3^ in size, with 3 T-type thermocouples fixed to the sample surface by polymeric glue used for thermal connection, to ensure a best transfer of heat to the thermocouples. The sensors are connected to a high-frequency (100 Hz) acquisition system (NI 9212 thermocouple input module for National Instruments CompactDAQ, Austin, TX, USA) controlled by software program, Labview (2020 community edition). We can consider for this set-up an intrinsic error in the temperature data of ±0.1 K.

## 3. Results and Discussion

**Figure 1:**
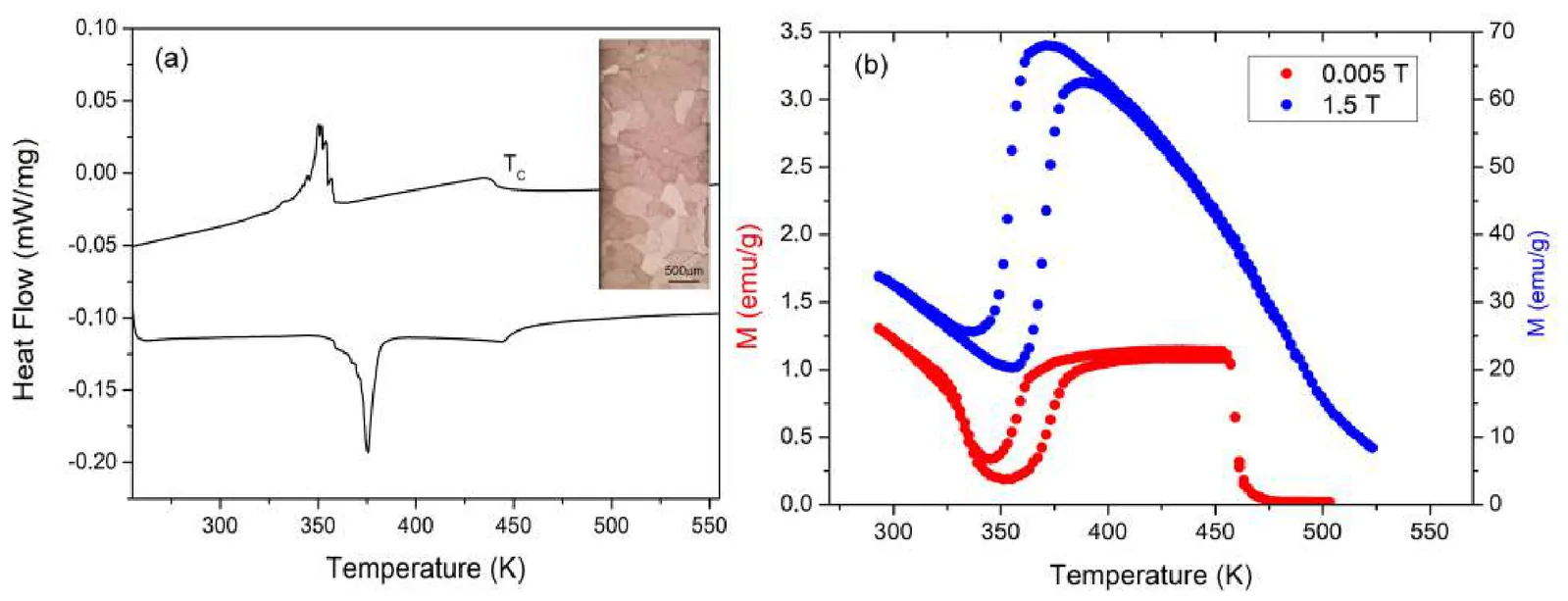
(**a**) Optical microscopy observation and DSC analysis (exothermal signal is up). (**b**) Magnetization curves vs. temperature at 0.005 T and 1.5 T.

Even if, at this stage of investigation, we are not focusing on studies of possible grain texture in the material, we have performed a first control of grain structure by optical microscopy. As reported in Figure 1a the alloy shows homogeneous grain size structure with an average size of 200 μm (see inset) and stable TMT with Ms = 358.4 K and Af = 382.1 K and a Curie temperature transition T_C_ at about 447 K. These values obtained by calorimetric analysis are in good agreement with those measured in the thermo-magnetic curve (Ms = 360.8 K; Af = 381.2 K, T_C_ = 454.6 K). As is clearly visible in Figure 1, the material shows no overlapping between MT and TMT, leading to a smaller magnetocaloric effect, with respect to what happens during transition between paramagnetic and ferromagnetic states. In this case, we only have transition between two ferromagnetic states into two different austenite and martensite crystal structures. In the present investigation, the application of magnetic fields does not bring appreciable changes of the above situation because possible overlapping of TMT and TM due to significant changes in transformation temperatures can occur for magnetic field values higher than 1.5 T (see Figure 1b).

The complete data of the DSC analysis are reported in Table 1 below.

The experimental values obtained for ΔH are in agreement with the literature values, and we use the calorimetric data as a principal reference for all entropy evaluations. From the calorimetric analysis, we can obtain some theoretical indication about the isothermal entropy change, ΔS, and adiabatic temperature change, ΔT_ad_, in caloric effects. Considering the following Equations (1) and (2),

(1) $${∆T}_{ad}^{th}=\frac{{∆S}_{tr}T}{C_{p}}=\frac{∆HT}{T_{0}C_{p}}$$

(2) $${∆S}_{DSC}^{th}=\frac{∆H}{T_{0}}$$

where ΔH is then enthalpy involved in the transition and the Cp is the specific heat measured by calorimetric analysis and T_0_ is defined as (Af + Ms)/2, we can obtain a first evaluation from a mere thermal point of view of ΔS as about 10.4 J/kgK and ΔT as about 5.5 K as caloric parameters related to TMT.

**Figure 2:**
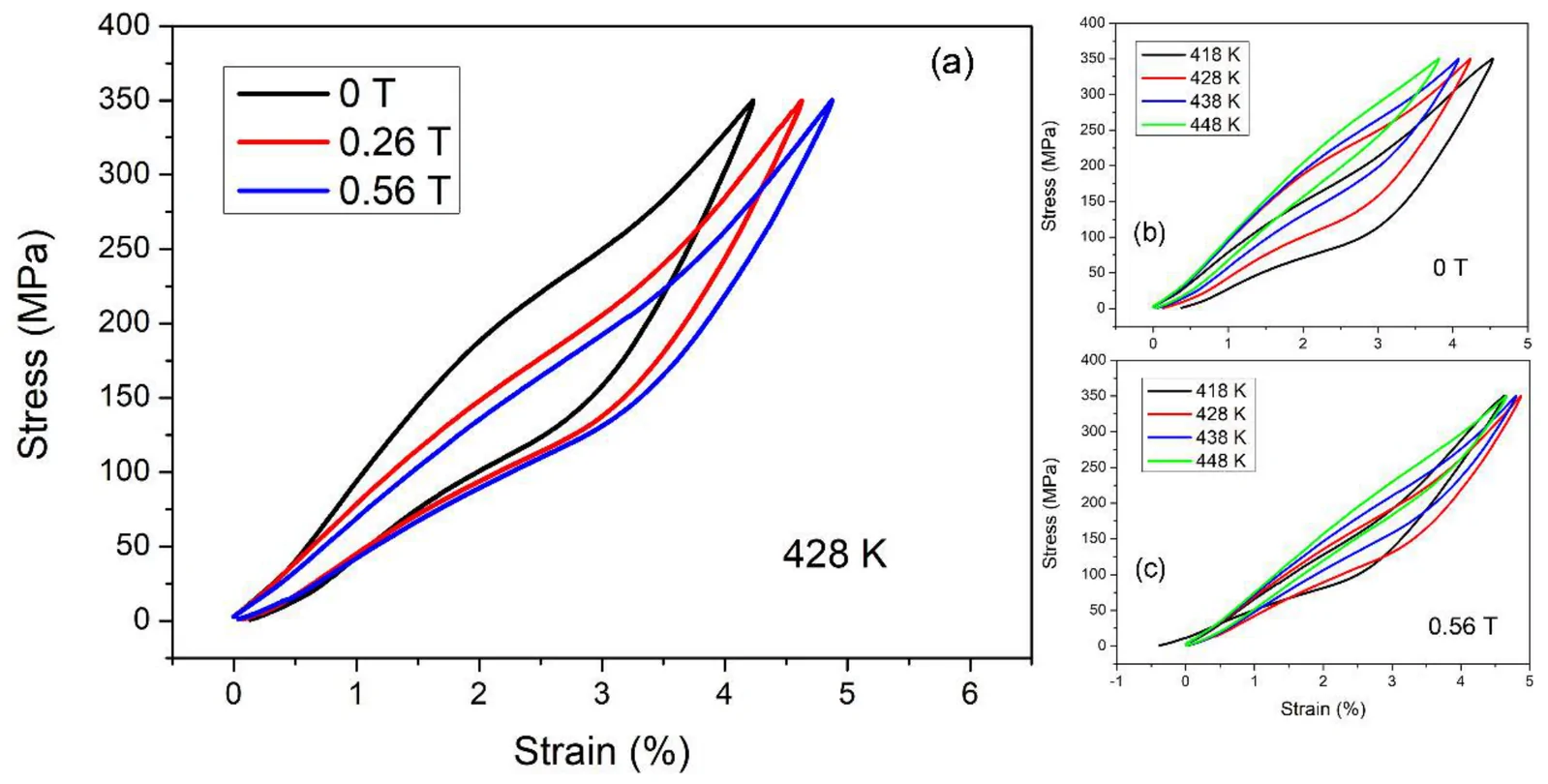
Mechanical curves registered in compression configuration: (**a**) stress–strain curves at 428 K vs. different magnetic field, (**b**) stress–strain curves at different temperatures at 0 T and (**c**) stress–strain curves at different temperatures at 0.56 T.

In Figure 2a, we report the stress–strain curves registered at 428 K at 0 T and two different fixed applied magnetic fields in addition to some examples of curves registered at different temperatures (Figure 2b,c). The pseudoelastic behavior reported shows similar aspects to those reported in our previous work on NiMnGaCu and NiMnCoTi [43,44].

When we apply stress and magnetic field simultaneously, the mechanical curve changes significantly, with the induction of detwinned and magnetically ordered martensite in the stress direction along the longitudinal magnetic field applied. This effect becomes more evident as the magnetic field is increased; we observe a general decrease in critical stress to induce martensite and the lowering of evidence of critical stress. We can highlight the main effects of simultaneous application of stress and magnetic field as follows:

(1) The increase in μ_0_H of the maximum strain achieved at fixed stress.
(2) The increase in quasi-elastic behavior of the martensite detwinning and the reduction in the visibility of the critical point corresponding to stress-induced martensite (SIM) and its detwinning plateau.
(3) The reduction in the critical stress for SIM.
(4) The reduction in the mechanical hysteresis.

This is the first indication that the magnetic ordering induced by the magnetic field acts as an overlapped longitudinal force that modifies the mechanical and therefore the elastocaloric response. We performed thorough mechanical analysis at different temperatures and at two values of magnetic field applied, to achieve an evaluation of the combined phenomena from an entropy change point of view, by using the Maxwell equation. It is a known fact that this approach could introduce some overestimation of ΔS, due to the presence of first-order phase transition, but it is also commonly accepted in the literature to use this approach as indicative information about ΔS. We refer to our previous work [45] for a more detailed discussion about this aspect, and in this work we present the results obtained by Maxwell elaboration in comparison with other kinds of calculation. We highlight the use of the CC coefficient to obtain an average value of ΔS, which is the principal reference of our discussion.

**Figure 3:**
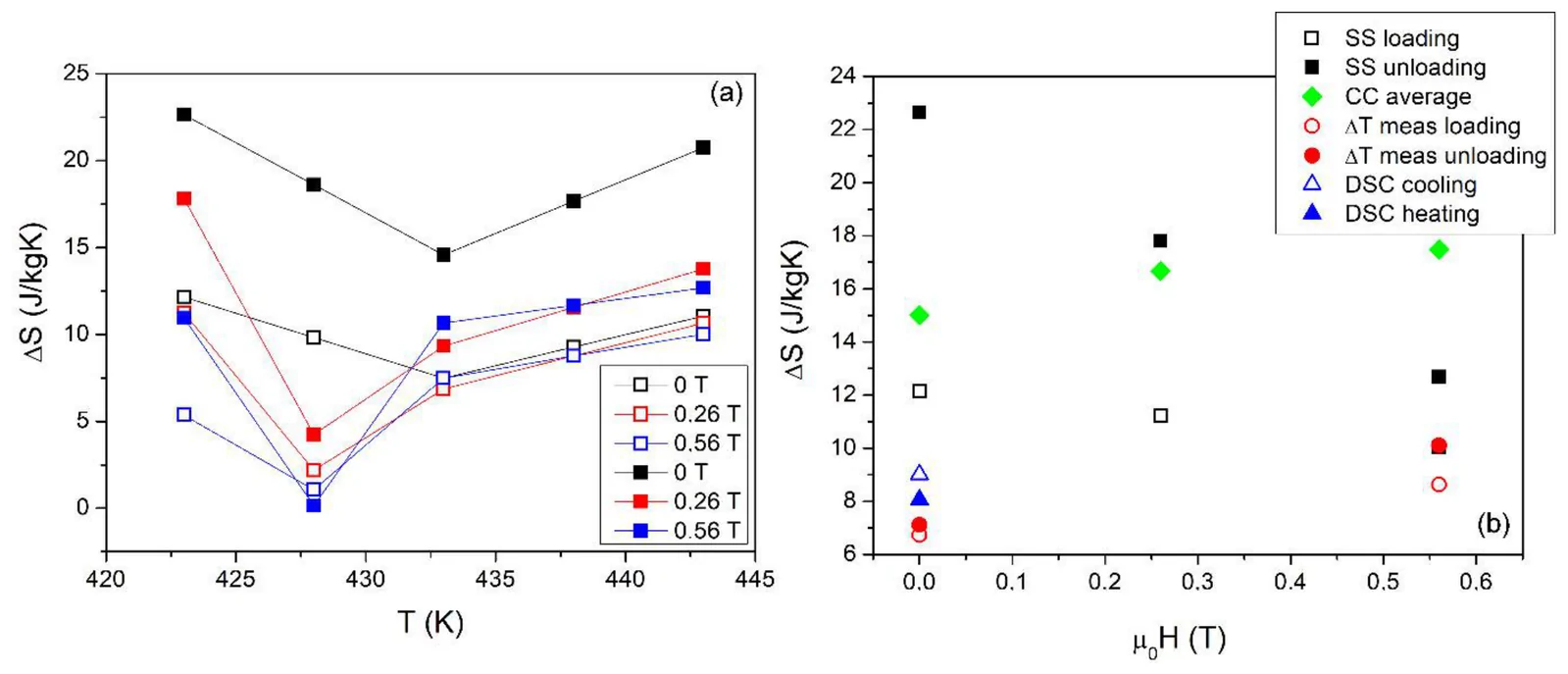
(**a**) ΔS values calculated by discrete integration of Maxwell equations of the curves registered in loading conditions (open square symbols) and unloading conditions (filled closed square symbols) at different temperatures and under magnetic field; (**b**) comparison of ΔS obtained from mechanical curves, calorimetric analysis and direct ΔT measurements.

**Figure 4:**
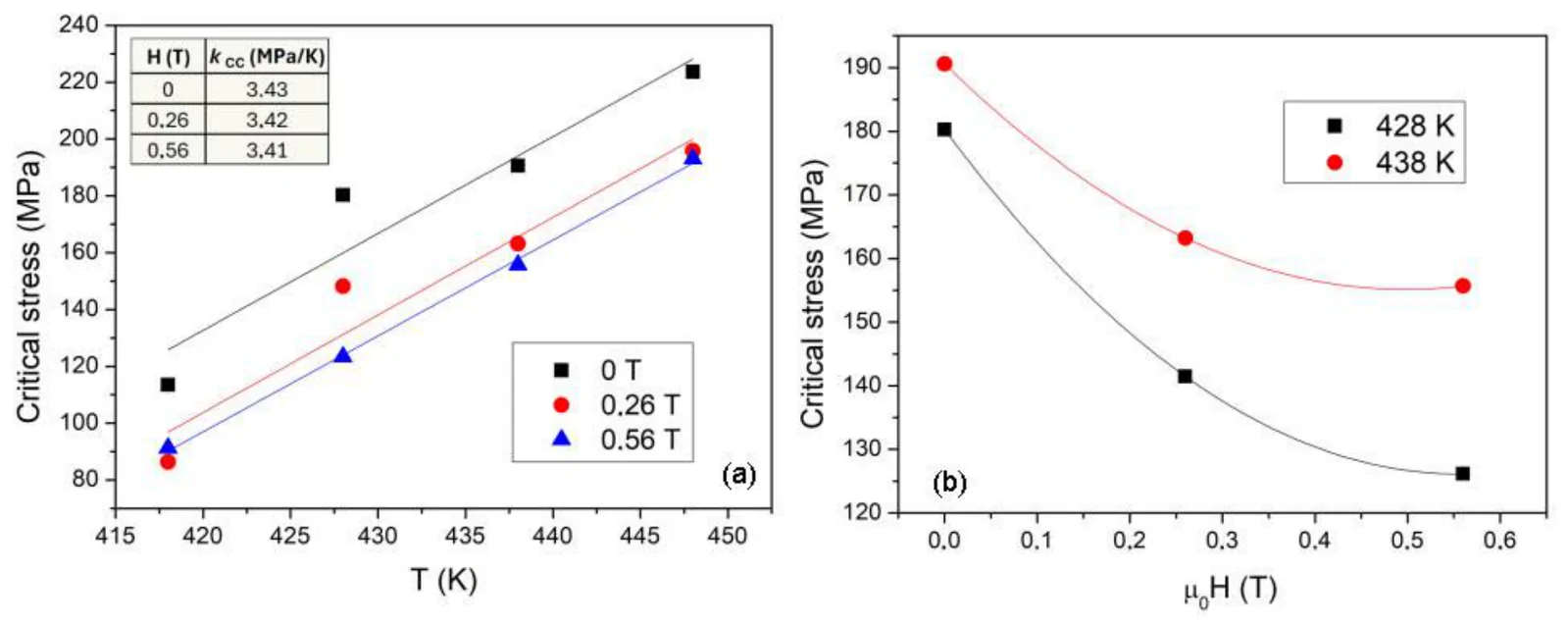
(**a**) On the left, critical stress behavior vs. temperature vs. magnetic field; in the inset, the Clausius Clapeyron coefficients (k_CC_) are calculated. (**b**) On the right, critical stress vs. magnetic field at two temperatures of registration of mechanical curves.

In Figure 3, we report the results obtained for ΔS values vs. temperature (Figure 3a) and vs. μ_0_H applied (Figure 3b). The entropy change shows maximum values for temperature near to Af (22.6 J/kgK at 423 K) at 0 T; the ones corresponding to the unloading step are generally higher than those of the loading part (12.2 J/kgK at 423 K), and only for the unloading data is a small decrease in ΔS value vs. μ_0_H observed. In fact, for temperatures above 430 K, the values related to loading are somewhat overlapped, and a reduced decrease appears for the unloading data. In Figure 4, we report the critical stress σc vs. temperature T at different magnetic fields μ_0_H (Figure 4a), compared to σc vs. μ_0_H at 428 and 438 K (Figure 4b).

The obtained values of Clausius Clapeyron (CC) coefficients k_CC_ are very stable, as shown in the inset in Figure 4a, and the decrease in the σc vs. μ_0_H does not look linear but hyperbolic (Figure 4b). Therefore, by considering an average evaluation of ΔS using the CC relationship, we obtain a quite constant behavior vs. μ_0_H, as represented in Figure 3, where the Clausius Clapeyron coefficients (k_CC_) are quite the same vs. μ_0_H, and the slight increase is related to the improvement of the maximum strain reached with the application of the magnetic field (from 15.1 to 17.48 J/kgK). It is interesting to note in the extended comparison in Figure 3b that the ΔS values obtained by using different experimental approaches are influenced by the transformation features associated with the measurements. For example, at 0 T it is noteworthy that the ΔS value obtained by DSC is lower than that obtained by the stress–strain test. This could be seen as a hint that the oriented detwinning in stress-induced martensite has a crucial contribution to the entropy change, with respect to twinned martensite registered in caloric measurements, namely a peculiar efficiency aspect of the elastocaloric effect. The comparison among the values calculated using stress–strain curves by the Maxwell equation or CC relationship are in good agreement, and they reflect the different aspects of the phenomenon considered in these elaborations. For discrete integration starting from the Maxwell equation, the chief parameter is the difference in stress plateau at different temperatures, and, as reported in Figure 2, one of the effects of magnetic field is to reduce mechanical hysteresis and the difference in stress level among the curves at different temperatures, inducing higher final strain. This explains the slight decrease in the single value at fixed temperature of maximum ΔS vs. μ_0_H. Such an effect disappears when averaging the behavior in the CC approximation. Finally, the direct experimental measurements of ΔT_ad_ through thermocouples overcome the limitations of elaboration of experimental isothermal mechanical or calorimetric data, since they are just related to the direct caloric effect under a magnetic field and quasi-adiabatic conditions. This experimental value can be elaborated as presented in Equation (1) to obtain an estimation of ΔH and then ΔS. As can be seen, the entropy change values obtained are underestimated but indicate an increase vs. μ_0_H, which also confirms, from an entropy point of view, the influence of the magnetic field, in agreement with the calculation using the CC relationship.

We can further observe that the effect of application of magnetic field is strictly related to changes in mechanical parameters like critical stress and total reached strain, and the effect on the order–disorder relationship guided by entropy change is reduced, likely because the magnetic transition coupled to elastocaloric effect is between two ferromagnetic structures. Therefore, it is reasonable to consider and evaluate the magnetic coupling as synergic, not only as a simple application at same time of the magnetic and mechanical force, but as a magnetostriction effect overlapping with the longitudinal compression stress applied, with consequent improved efficiency of the elastocaloric effect. Moreover, the use of only two discrete values of magnetic field hinders the possibility to use double integration for the approach of evaluation of magnetic and mechanical entropy changes.

**Figure 5:**
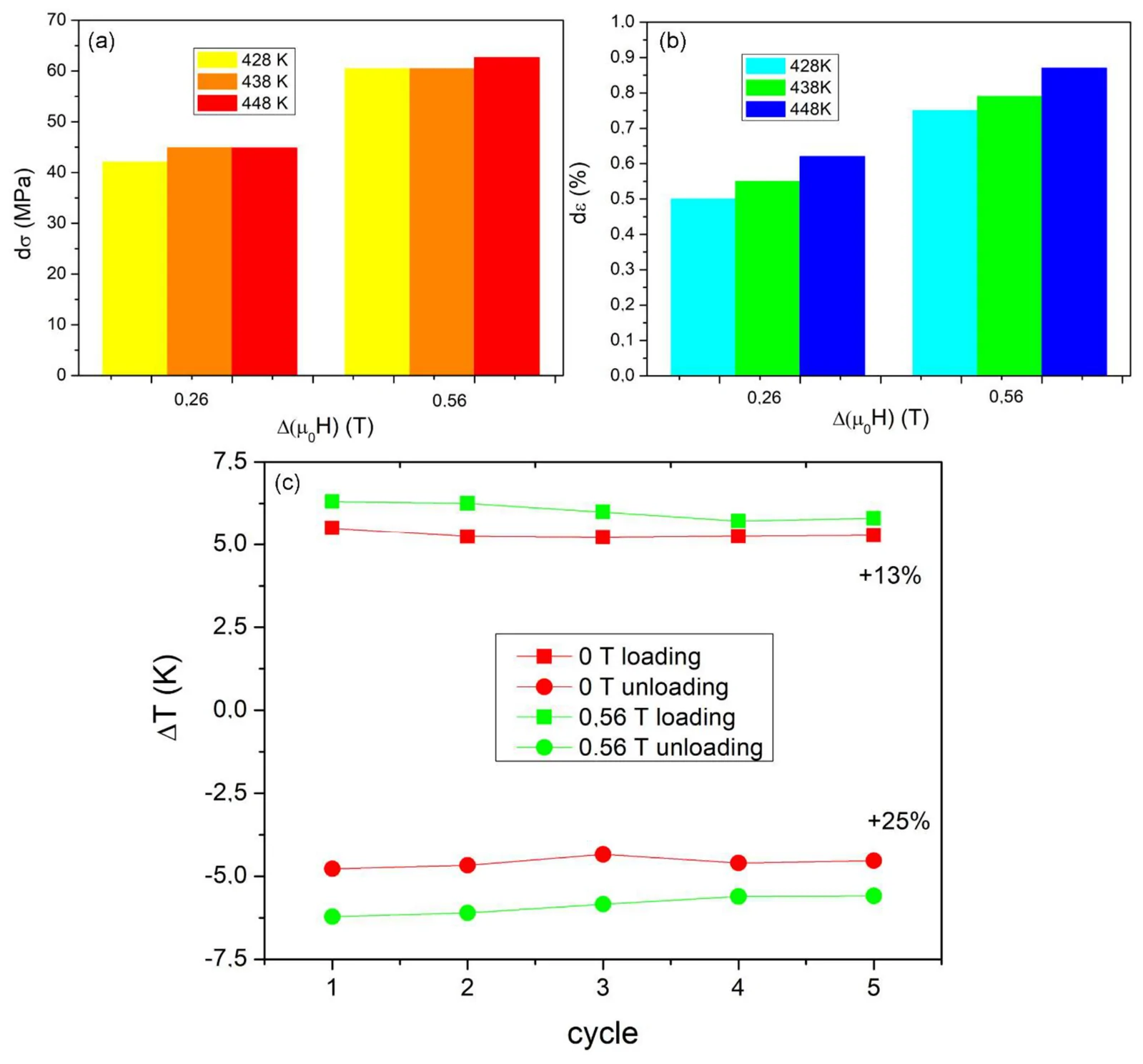
(**a**) Magnetostrictive stress induced by the magnetic field at three different temperatures; (**b**) magnetostrictive strain induced by the magnetic field at three different temperatures; (**c**) ΔT direct measures under magnetic field.

The ad hoc set-up developed for these simultaneous magneto-mechanical measurements reduced space around the sample, and it is impossible to use other accessories such as strain gauges or similar devices to obtain supplementary data on the deformation process. Therefore, at this first step of investigation, we cannot directly measure the magnetostrictive effect, but we can indirectly elaborate it by comparison and elaboration of mechanical curves. Therefore, the results proposed are estimated values of effective additional stress which could be associated with a magnetostrictive effect. In Figure 5a,b, we present the indirect magnetostriction parameter obtained at different temperatures, representing, in terms of stress and strain induced, the direct effect of the magnetic field application. The magnetostriction contribution is not measured experimentally, but it is deduced by comparison of the mechanical curves at different strains. In this way it is possible to obtain this evaluation and consider the fixed magnetic field coupled to the stress field as a fixed amount of stress adding to the stress applied by the experimental compression set-up. In fact, the magnetostrictive strain is calculated from the subtraction of the maximum strain of the mechanical curves at fixed (max) stress. We consider the approximation of linear one-dimensional magnetostriction because in our work stress and magnetic fields have the same longitudinal direction in the sample, and the strain is calculated in the same direction. The analysis presented in Figure 5a,b gives a first linear approximation of stress and strain induce by magnetic field, which overlaps with the tensile stress and strain. The values are calculated by comparison of the mechanical curves obtained at different values of μ_0_H and temperatures, at fixed strain or stress. The maximum strain/stress induced is quite constant at different temperatures, and it correctly increases vs. the magnetic field (from 45 MPa to 60 MPa, and from 0.6% to 0.85%). This alloy therefore exhibits giant magnetostrictive strain, which is typical of FeSMAs.

The last experimental part presented is the independent control of the effect of overlapping of the magnetic and mechanical fields. The direct measurements of the ΔT_ad_ expressed by the material is the final real parameter, which confirms the improvement of the multicaloric effect by this coupling approach. Then finally, in Figure 5c we report on the results of the direct measurements of ΔT_ad_ at 0 T and 0.56 T. The aim of these measures is to give first experimental control of the critical aspect of combination of magnetic and mechanical contribution; therefore, it is limited to the first stable series of quasi-adiabatic compression cycles. Preliminary results over 10 cycles show no significant degradation of ΔT_ad_, but long-term stability requires further investigation. The values registered consider the total caloric effect represented by the amount of difference in temperature expressed by the material, and it is the first straightforward parameter affected by the overlapping of the magnetic and mechanical fields’ action. As can be seen in the figure, the value registered at 0 T is in good agreement with the theoretical value previously reported and obtained from the DSC analysis. The improvement in elastocaloric effect and in ΔT_ad_, due to the application of the magnetic field, is reflected in an increase of 13% in loading and 25% in unloading measurements, where, in agreement with the previous analysis, we have the best efficiency of the caloric phenomenon.

## 4. Conclusions

In this work, an ad hoc experimental set-up developed to perform mechanical measurements under discrete magnetic field values at different temperatures is presented. Even if in this first step we apply only two discrete values of magnetic field, i.e., 0.26 T and 0.56 T, these first measurements are useful to give an important indication of magneto-mechanical coupling in the NiMnGaCo alloy. Indeed, both isothermal mechanical tests and quasi-adiabatic measurements were performed in compression configuration under a magnetic field. The elaboration of the isothermal mechanical curves gives us the possibility to evaluate the entropy change and the adiabatic temperature change and their general improvement, due to the application of the magnetic field. The effect of the simultaneous application of both mechanical stress and the magnetic field seems not simply combined, but they “help” each other in a synergic way. Indeed, a significant improvement in the elastocaloric effect is observed when the magnetic field is applied, because it has the effect of reducing critical stress and increasing total strain in the mechanical response of the material. Moreover, it is noteworthy that also at these relatively low values of magnetic field we can observe the improvement of the caloric properties of NiMnGaCo due to magnetocaloric effect. The analysis of the magnetic field’s contribution, as the magnetostrictive effect, to mechanical stress drives an increase in elastocaloric efficiency. Then the magnetostrictive approach is useful for a direct quantitative evaluation of this enhancement. The improvement in elastocaloric effect and in adiabatic temperature change, due to the application of the magnetic field, is reflected in an increase of 13% in loading and 25% in unloading measurements. The next step of this investigation will be related principally to two aspects: the use of more discrete values of magnetic field, by combination of different kinds of permanent magnets and the application of a new set-up with a transversal direction of the magnetic field. Moreover, a refined numerical approach will be provided to better understand the overlapping contribution of the magnetic and mechanical fields.

## Figures and Tables

**Table 1:** Data of the DSC analysis.

Ms	Mf	H Cooling	As	Af	H Heating
358.4 K	331.5 K	3.518 J/g	367.9 K	382.1 K	3.156 J/g

## Data Availability

The original contributions presented in this study are included in the article. Further inquiries can be directed to the corresponding author.
